# Safety and efficacy of radiotherapy/chemoradiotherapy combined with immune checkpoint inhibitors for non-small cell lung cancer: A systematic review and meta-analysis

**DOI:** 10.3389/fimmu.2023.1065510

**Published:** 2023-03-13

**Authors:** Jing Wu, Tingting Ni, Rong Deng, Yan Li, Qin Zhong, Fei Tang, Qi Zhang, Chunju Fang, Yingbo Xue, Yan Zha, Yu Zhang

**Affiliations:** ^1^ Department of Medical Oncology, Guizhou Province People’s Hospital, Guiyang, China; ^2^ Department of Nephrology, Guizhou Provincial People's Hospital, Guiyang, China; ^3^ National Health Commission Key Laboratory of Pulmonary Immune-Related Diseases, Guizhou Province People's Hospital, Guiyang, Guizhou, China

**Keywords:** immune checkpoint inhibitors, radiation therapy, non-small cell lung cancer, efficacy, safety, meta-analysis

## Abstract

**Background:**

It is now widely accepted that radiotherapy (RT) can provoke a systemic immune response, which gives a strong rationale for the combination of RT and immune checkpoint inhibitors (ICIs). However, RT is a double-edged sword that not only enhances systemic antitumor immune response, but also promotes immunosuppression to some extent. Nevertheless, many aspects regarding the efficacy and safety of this combination therapy remain unknown. Therefore, a systematic review and meta-analysis was performed in order to assess the safety and efficacy of RT/chemoradiotherapy (CRT) and ICI combination therapy for non-small cell lung cancer (NSCLC) patients.

**Methods:**

PubMed and several other databases were searched (according to specific criteria) to find relevant studies published prior to the 28^th^ of February 2022.

**Results:**

3,652 articles were identified for screening and 25 trials containing 1,645 NSCLC patients were identified. For stage II-III NSCLC, the one- and two-year overall survival (OS) was 83.25% (95% confidence interval (CI): 79.42%-86.75%) and 66.16% (95% CI: 62.3%-69.92%), respectively. For stage IV NSCLC, the one- and two-year OS was 50% and 25%. In our study, the pooled rate of grade 3-5 adverse events (AEs) and grade 5 AEs was 30.18% (95% CI: 10.04%-50.33%, I^2^: 96.7%) and 2.03% (95% CI: 0.03%-4.04%, I^2^: 36.8%), respectively. Fatigue (50.97%), dyspnea (46.06%), dysphagia (10%-82.5%), leucopenia (47.6%), anaemia (5%-47.6%), cough (40.09%), esophagitis (38.51%), fever (32.5%-38.1%), neutropenia (12.5%-38.1%), alopecia (35%), nausea (30.51%) and pneumonitis (28.53%) were the most common adverse events for the combined treatment. The incidence of cardiotoxicity (0%-5.00%) was low, but it was associated with a high mortality rate (0%-2.56%). Furthermore, the incidence of pneumonitis was 28.53% (95% CI: 19.22%-38.88%, I^2^: 92.00%), grade ≥ 3 pneumonitis was 5.82% (95% CI: 3.75%-8.32%, I^2^: 57.90%) and grade 5 was 0%-4.76%.

**Conclusion:**

This study suggests that the addition of ICIs to RT/CRT for NSCLC patients may be both safe and feasible. We also summarize details of different RT combinations with ICIs to treat NSCLC. These findings may help guide the design of future trials, the testing of concurrent or sequential combinations for ICIs and RT/CRT could be particularly useful to guide the treatment of NSCLC patients.

## 1 Introduction

Non‐small cell lung cancer (NSCLC) is the leading cause of cancer mortality in the world (1). Immune Checkpoint Inhibitors (ICI) for NSCLC have become a common treatment strategy as numerous clinical trials have demonstrated their clinical benefits (2–4). Radiotherapy (RT) has been the predominant conventional local treatment for both locally advanced and metastasis NSCLC patients. It can be used for either curative or palliative purposes. Palliative RT can not only improve progression-free survival (PFS) and overall survival (OS) for metastasis NSCLC patients but also improve the patient’s quality of life (QOL) by relieving or avoiding the occurrence of symptoms (5–7). On the other hand, more aggressive treatments can avoid symptoms from local progression or delay initiation of a new systemic therapy (8). Mole (9) reported the abscopal effect in 1953, where radiation at one site may lead to tumor regression in both distal and distal non-irradiated sites. In recent years, RT has been described to increase the effects of ICIs on systemic antitumor responses by affecting almost all steps of the cancer-immunity cycle. RT can convert “cold” tumors that are typified by low immune cell infiltration into “hot” tumors with lymphocytic infiltration (10). The immunomodulatory effects associated with RT provide the premise underlying ICI response and a combination therapeutic strategy. Indeed, RT and ICI (RT&ICI) combination therapies have been proven to successfully treat patients with many kinds of cancers (11–13). Shaverdian et al. (14) found that patients with advanced NSCLC had longer PFS and OS when administered a therapeutic regimen that combined RT&ICIs. Similarly, Formenti et al. (15) found that patients with NSCLC that was previously unresponsive to ICI therapy experienced longer OS when treated with a RT&ICI combination.

RT can prove to be a double-edged sword that not only enhances systemic antitumor immune responses; but can also lead to the promotion of immunosuppression. For example, RT can promote immune response efficacy *via* T cell activation (16); yet it can cause lymphopenia and thereby reduce the efficacy of combination therapies (17). On one hand, RT may induce tumor-associated neutrophils to exhibit antitumor properties through interferon-β; on the other hand, pro-tumor properties may be induced *via* transforming growth factor-β (10). Some studies have also found that RT may increase myeloid-derived suppressor cell abundance, which can contribute to tumor progression in certain human organs or tissues (18). However, other studies have found that RT may promote dendritic cell mediated immune system efficacy (10, 19).

The potential for RT&ICI associated toxicity is also noteworthy. The most serious adverse effects include radiation-induced lung injury and radiation-induced heart disease, the latter of which can present with cardiomyopathy, conduction system abnormalities and coronary artery disease. The incidence of potentially lethal ICI-associated pneumonitis and cardiotoxicity is 3% to 5% (20) and 3.1% (21), respectively. Moreover, some preclinical studies have reported increased toxicity (especially pulmonary toxicity) when ICIs were combined with RT (22, 23). In a post-hoc analysis of the phase I KEYNOTE-001 trial, patients who had received prior chest RT developed more pulmonary toxicities when compared to patients without prior chest RT (13% vs. 1%, *P*=0.046) (14). Botticella et al. (24) also observed that patients receiving combination therapy were more likely to develop grade ≥3 pneumonitis when compared to patients receiving ICIs alone (16.7% vs. 2.4%, *P*=<0.001). Additionally, radiation recall pneumonitis localized within a previously irradiated area may occur when using ICIs (25). Hence, the aforementioned adverse events raise substantial concerns for the overlapping toxicity associated with RT&ICIs.

The efficacy and safety of RT/chemoradiotherapy (CRT) when combined with ICIs remains controversial, with the selection of ICIs needing further exploration and optimization. This is also true for aspects regarding the accompanying RT, including: ICI agent selection, treatment sequencing, RT dose, fractionation and irradiated site applicability. Therefore, a systematic review and meta-analysis to elucidate RT&ICI combination therapy efficacy and toxicity for NSCLC was performed and described herein.

## 2 Materials and methods

### 2.1 Search strategy and selection criteria

This systematic review and meta-analysis was conducted using the Preferred Reporting Items for Systematic Review and Meta-analysis statement (26). Ethical approval was not required for this study since the data included was obtained exclusively from previously published sources.

PubMed, Web of Science and the Cochrane Library were searched to identify literature in English language journals published prior to the 28^th^ of February 2022, without a lower date boundary. The following search terms were used: 1) “non-small cell lung neoplasm (s)/cancer (s)/carcinoma (s)” or “NSCLC”. 2) “radiotherapy” or “radiation therapy” or “radiation treatment” or “irradiation” or “SABR” or “stereotactic ablative radiotherapy” or “SBRT” or “stereotactic body radiation therapy” or “SRS” or “stereotactic radiosurgery” or “SRT” or “stereotactic radiotherapy” or “radio-chemotherapy” or “chemoradiotherapy”. 3) “immunotherapy” or “immune checkpoint inhibitors” or “checkpoint inhibitor “ or “checkpoint blockade” or “programmed cell death 1 receptor” or “programmed cell death 1 ligand 1” or “programmed death-1” or “PD-1” or “programmed death ligand-1” or “PD-L1” or “cytotoxic T lymphocyte-associated antigen-4 (CTLA-4) antigen” or “anti-CTLA-4” or “anti-PD-1” or “anti-PD-L1” or “CTLA-4” or “Durvalumab” or “Imfinzi” or “ MEDI-4736” or “Atezolizumab” or “ MPDL3280A” or “Tecentriq” or “RO5541267” or “RG7446” or “Pembrolizumab” or “Lambrolizumab” or “Keytruda” or “SCH 900475” or “MK-3475” or “Nivolumab” or “Opdivo” or “Ono-4538” or “MDX-1106” or “BMS-936558” or “Nivo” or “Avelumab” or “Barvencik” or “MSB0010718C” or “Toripalima” or “Tislelizumab” or “Camrelizumab” or “Sintilimab” or “Tremelimumab” or “Ipilimumab” or “Cemiplimab” or “Libtayo” or “PDCD1” or “CD274”.The comprehensive search string is available as **Supplementary Materials Table 1**.

Studies that met the following criteria were included in the meta-analysis: 1) describe participants with histologically confirmed lung cancer; 2) original papers describing human clinical trials that reported the outcomes for CRT/RT combined with ICIs; 3) prospective or retrospective study; 4) outcomes included treatment safety and efficacy; 5) published in English.

Conference abstracts, case reports, comments, reviews, animal studies, and mechanistic studies were excluded. Studies that did not have sufficient data (missing clinical safety data and efficacy data) or unclear descriptions (poorly described trials or did not accurately describe trial results) were also excluded. When articles described the same study population, only the most recent or complete analysis was included. Disagreements related to article selection were resolved in a discussion with all the authors of this study.

### 2.2 Data extraction

The data was extracted by two independent investigators, including: first author name, time of publication, country, number of cases, pathological subgroups, type of study, treatment regimens, radiotherapy type and dose, treatment characteristics including timing of ICI therapy and rates of pneumonitis and treatment related deaths. Any discrepancies were resolved through group discussions until a consensus was reached. The revised Cochrane Risk of Bias tool for randomized trials (27) and Risk of Bias in Non-randomized Studies of Interventions tool (28) were conducted to appraise the quality of randomized controlled trials and non-randomized trials, respectively.

### 2.3 Statistical analysis

The random effects model was used for the meta-analysis with the “meta” package implemented in R (version 4.1.2, R Foundation for Statistical Computing). A 95% confidence interval (CI) was used, and Cochran’s Q and I^2^ statistics were used to assess heterogeneity. 0%, 25%, 50% and 75% of I^2^ represented no, low, moderate and high heterogeneity, respectively. Meta-regression was considered inappropriate due to the insufficient number of studies (<10). Subgroup and sensitivity analyses were performed to explore study heterogeneity and to determine the impact of each individual study. Publication bias was estimated using the Begg’s and Egger’s test. *P=*<0.05 was used as the threshold for considering statistical significance.

## 3 Results

**Figure 1:**
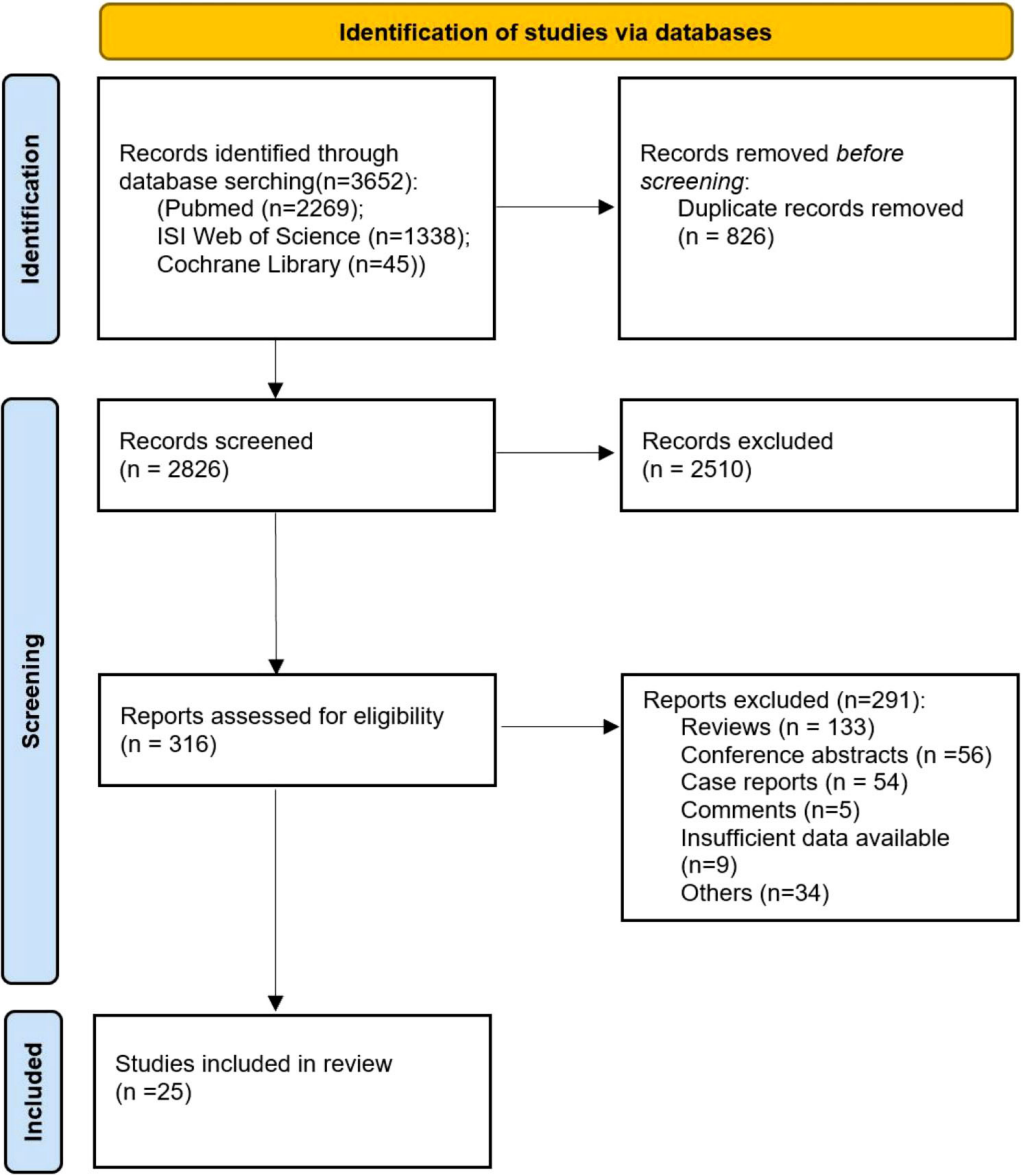
Flow diagram of included and excluded studies.

**Figure 2:**
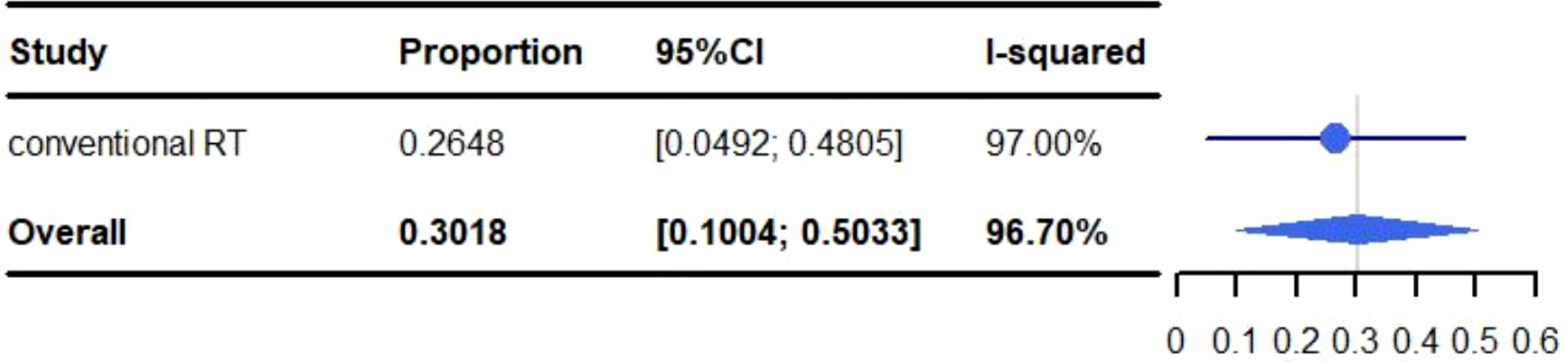
Meta-analysis and forest plots for non-small cell lung cancer patients treated with radiotherapy/chemoradiotherapy and immune checkpoint inhibitors who experienced grade 3-5 adverse events.

The search process (**Figure 1**) enabled the identification of twenty-five trials (**Table 1**) for this systematic review and meta-analysis (11, 14, 29–51). The twenty-five trials consisted of twenty-four trials non-randomized trials (11, 14, 29, 31–51) and one randomized controlled trial (30). Thirteen trials were conducted in North America (11, 14, 29, 31, 33, 34, 39, 43, 45, 46, 49–51), nine in Asia (32, 35–38, 40–42, 44), and three in Europe (30, 47, 48). Of the twenty-five studies included, nine were prospective studies (14, 29, 30, 39, 45, 46, 48–50) and sixteen were retrospective studies (11, 31–38, 40–44, 47, 51). Two trials utilized conventional RT and stereotactic body radiotherapy (SBRT) (46, 51), two focused upon SBRT (11, 50) and twenty-one trials used conventional RT (14, 29–45, 47–49). Durvalumab (30–38, 47), pembrolizumab (14, 29, 46, 49), nivolumab (40, 41, 48), ipilimumab (45), and atezolizumab (39) were used in nine, four, three, one and one study (ies), respectively; pembrolizumab and nivolumab (43, 44, 50) were used in three studies. ICIs were administered after RT in eighteen studies (11, 14, 29–38, 40–44, 50), prior to RT in one study (45), concurrently with CRT in three studies (11, 46, 50), whereas two studies examined both concurrent and sequential administration of RT with ICIs (48, 49). A total radiation dose of < 60 Gy (35, 40, 46, 49) was administered in four studies and a total radiation dose of >=60 Gy (34, 39, 48) was prescribed in three studies. Both randomized (30) and non-randomized trials (11, 14, 29, 31–51) were judged to be at moderate risk of bias (**Supplementary Materials Table 2, Table 3** and **Figures 1**, **2**). A total of 1,645 patients were included in the aforementioned trials.

**Table 1:** Main study characteristics.

Author	Year	Nation	N	Study Type	RT dose/fraction	ICIs Sequencing	ICIs Type	Intervention Period	Toxicity ≥ Grade 3 (%)	Grade 5 Toxicity (%)	All Grade Pneumonitis (%)	Pneumonitis≥ Grade 3 (%)	Grade 5 Pneumonitis (%)	
Shukla	2021	USA	92	Prospective	5940-6660c Gy	Sequential	Pemb (PD-1)	12 months	NR	NR	18.5%	4.3%	1.1%	
Shaverdian	2017	USA	24	Prospective	NR	Sequential	Pemb (PD-1)	NR	NR	NR	8.4%	4.2%	0%	
Finn	2020	UK	475	Prospective	<6000cGy (8%) 6000-6600c Gy (86%) >6600cGy (6%)	Sequential	Dura (PD-L1)	12 months	37.1%	4.4%	33.9%	4.6%	1.1%	
LeClair	2022	USA	83	Retrospective	<6000cGy (4%) 6000cGy (48%) >6000cGy (34%) NR (16%)	Sequential	Dura (PD-L1)	average of 13.9 (range 1-47) doses	NR	NR	25.3%	7.2%	1.2%	
Landman	2021	Israel	39	Retrospective	Mean dose 6990cGy	Sequential	Dura (PD-L1)	average of 21 (range 1-26) doses	NR	NR	15%	15%	3%	
Aredo	2021	USA	13	Retrospective	5000cGy (7.7%) 6000cGy (46.2%) 6600cGy (46.2%)	Sequential	Dura (PD-L1)	average of 6 (range 4-14) doses	41.7%	0%	23%	15.4%	0%	
Hassanzadeh	2020	USA	34	Retrospective	6000-7000cGy/30-35f	Sequential	Dura (PD-L1)	average of 8.5 (range 1-26) doses	NR	NR	26.5%	5.9%	0%	
Miura	2020	Japan	41	Retrospective	6000Gy/30f (98%) 5400Gy/25f (2%)	Sequential	Dura (PD-L1)	NR	7.3%	0%	61.0%	2.4%	0%	
Jung	2020	Korea	21	Retrospective	5400-6600c Gy	Sequential	Dura (PD-L1)	NR	NR	NR	81%	14.3%	0%	
Inoue	2020	Japan	30	Retrospective	NR	Sequential	Dura (PD-L1)	NR	NR	NR	73.3%	0%	0%	
Chu	2020	China	29	Retrospective	<6000cGy (63.9%) 6000-6600c Gy (25.8%) >6600cGy (9.7%)	Sequential	Dura (PD-L1)	2.8 (1.8–3.7) months	13.8%	0%	17.2%	6.9%	0%	
Lin	2020	USA	40	Prospective	6000-6600cGy	Sequential (25%)/Concurrent and Sequential (75%)	Atez (PD-L1)	12 months	80%	10%	25%	2.5%	0%	
Yamaguchi	2019	Japan	40	Retrospective	5000-6000cGy (60%) 800-3000cGy (40%)	Sequential	Nivo (PD-1)	NR	NR	NR	20%	NR	NR	
Tamiya	2017	Japan	50	Retrospective	NR	Sequential	Nivo (PD-1)	NR	NR	NR	22%	NR	NR	
Jang	2021	Korea	51	Retrospective	4600–7300Gy	Sequential	Dura (PD-L1) (68.6%) Pemb (PD-1) (11.8%) Nivo (PD-1) (9.8%) Atez (PD-L1) (9.8%)	average of 9 (range 2-24) doses	NR	NR	52.9%	5.9%	0%	
Barrón	2020	Colombia	40	Retrospective	<6000cGy (52.5%) ≥6000cGy (47.5%)	Sequential	Pemb (PD-1) or Nivo (PD-1)	NR	NR	NR	40%	10%	0%	
Amino	2020	Japan	20	Retrospective	5400-6600cGy	Sequential	Pemb (PD-1) (5%) Nivo (PD-1) (95%)	NR	5%	0%	5%	0%	0%	
Boyer	2016	USA	16	Prospective	3600-7400cGy	Before	Ipi (CTLA4)	2 doses	0%	0%	0%	0%	0%	
Bruni	2021	Italy	155	Retrospective	<6000cGy (7.7%) 6000-6600c Gy (82.0%) >6600cGy (10.3%)	Concurrent (58.7%) Sequential (41.3%)	Dura (PD-L1)	NR	NR	NR	17.4%	2.5%	0%	
Peters	2021	Switzerland	77	Prospective	6600Gy/33f	Concurrent and Sequential	Nivo (PD-1)	12 months	NR	NR	44.2%	11.7%	1.3%	
Jabbou	2020	Canada	21	Prospective	6000Gy/30f (95%) 4000cGy/20f (5%)	Concurrent and Sequential	Pemb (PD-1)	12 months	NR	4.8%	33%	10%	4.7%	
SBRT														
Bestvina	2021	USA	37	Prospective	4500cGy/3f	Concurrent/Sequential	Pemb (PD-1) Nivo (PD-1)	2 years	56.8%	8.1%	`24.3%	24.3%	0%	
Chen	2020	USA	33	Retrospective	5000cGy/4f 6000cGy/10f	Concurrent/Sequential	CTLA-4+PD-1/PD-1	4 doses/NR	NR	0%	12,1%	12.1%	0%	
RT & SBRT														
Voong	2019	USA	100	Retrospective	NR	NR	PD-1 PD-L1	NR	NR	NR	19%	NR	NR	
Welsh	2020	USA	80	Prospective	5000cGy/4f/4500cGy/30f	Concurrent	Pemb (PD-1)	32 doses	NR	NR	5%	3.75%	0%	

N, Number of patients; ICIs, Immune checkpoint inhibitors; Nivo, Nivolumab; Ipi, Ipilimumab; PD-1, Programmed death-1; CTLA-4, Cytotoxic T lymphocyte-associated antigen-4; USA, United States of America; Pemb, Pembrolizumab; NR, Not reported; UK, United Kingdom; Dura, Durvalumab; PD-L1, Programmed death ligand-1; Atez, Atezolizumab.

### 3.1 Efficacy and survival

The trials included in this study had different primary efficacy variables, which prevented the performance of an efficacy based meta-analysis. The objective response rate (ORR) was 25.8%-73.4% for II-III stage NSCLC and 45%-45.9% for IV stage NSCLC. The one-year to five-year PFS and OS for II-III stage NSCLC and IV stage NSCLC were shown in **Table 2**.

**Table 2:** Non-small cell lung cancer patient survival following treatment with ICIs and RT.

	Stage II-III NSCLC	Stage IV NSCLC
1-year PFS	56.39% (95% CI: 50.66%-62.03%, I^2^:39.4%)	—
2-year PFS	43.58%-45%	—
3-year PFS	39.7%	—
4-year PFS	35.0%	—
5-year PFS	33.1%	—
1-year OS	83.25% (95% CI: 79.42%-86.75%, I^2^:17.6%)	50%
2-year OS	66.16% (95% CI: 62.30%-69.92%, I^2^:0.0%)	25%
3-year OS	56.7%	—
4-year OS	49.7%	—
5-year OS	42.9%	—

ICIs, Immune checkpoint inhibitors; RT, Radiotherapy; PFS, Progression Free Survival; OS, Overall Survival; NSCLC, non-small cell lung cancer.

### 3.2 Adverse reactions

**Figure 3:**
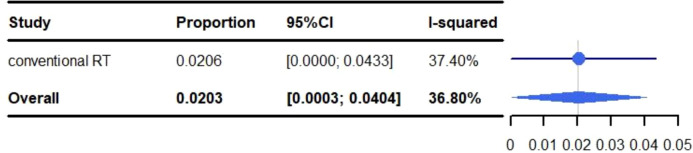
Meta-analysis and forest plots for non-small cell lung cancer patients treated with radiotherapy/chemoradiotherapy and immune checkpoint inhibitors who experienced grade 5 adverse events.

All adverse events are described in **Table 3**. The pooled rate for grade 3-5 adverse reactions was 30.18% (95% CI: 10.04%-50.33%, I^2^: 96.7%) and 2.03% (95% CI: 0.03%-4.04%, I^2^: 36.8%) for grade 5 adverse events (**Figures 2**, **3**). The most common adverse events included: fatigue (50.97%), dyspnea (46.06%), dysphagia (10%-82.5%), leucopenia (47.6%), anaemia (5%-47.6%), cough (40.09%), esophagitis (38.51%), fever (32.50%-38.10%), neutropenia (12.5%-38.1%), alopecia (35%), nausea (30.51%) and pneumonitis (28.53%). Most events were grade 1-2. The most common grade 3-5 events included: inflection (0.2%-20%), cough (0%-15%), gastric hemorrhage (2.5%-8.1%), elevated liver enzymes (7.64%), neutropenia (2.5%-9.5%), dehydration (0%-7.5%), pneumonitis (5.82%) and adrenal insufficiency (5.40%). Of the patients with elevated liver enzymes, adrenal insufficiency and gastric hemorrhage, all the events were categorized as grade 3-5. The most common grade 5 adverse reactions were pneumonitis (0%-4.76%), gastric hemorrhage (0%-2.70%) and myocardial infarction (0.21%-2.56%). For pulmonary adverse events, the grade 1-5 adverse reactions accounted for 0.21%-46.06%, and the most common effects included: dyspnea (46.06%), cough (40.09%), esophagitis (38.51%) and pneumonitis (28.53%). The grade 3-5 adverse reactions accounted for 0%-15.00%, and the most common side effects included: pneumonitis (5.82%) and cough (0%-15.00%). The grade 5 adverse reactions included: pneumonitis (0%-4.76%), emphysema and respiratory failure (0.2%-0.21%), hemoptysis (0%-0.2%) and dyspnea (0%-0.2%). The incidence of hemoptysis, pulmonary embolism, emphysema and respiratory failure, was relatively low; but were grade 3-5 events. The mortality associated with hemoptysis, pulmonary embolism, emphysema and respiratory failure was 0%-0.21%. It is worth noting that whilst the incidence of cardiotoxicity (0%-5%) was low, it was associated with a high mortality rate (0%-2.56%). The grade 3-5 adverse reactions accounted for 0%-2.7%, with the most common effects including: acute coronary syndrome (2.5%), heart failure (2.5%), atrial fibrillation (0%-2.70%) and myocardial infarction (0.21%-2.56%). The grade 5 adverse reactions observed during the trials were: myocardial infarction (0.21%-2.56%), cardiac arrest (0.42%), cardiomyopathy (0.21%), cardiopulmonary failure (0.21%), aortic dissection (0.21%) and right ventricular failure (0.21%). We identified that myocardial infarction was adverse event associated with the highest risk of mortality.

**Table 3:** Adverse events experienced by non-small cell lung cancer patients following treatment with ICIs and RT.

	Grade 1-5	Grade 3-5	Grade 5
Pulmonary			
Pneumonitis	28.53% (95% CI: 19.22%-38.88%, I^2^: 92.00%)	5.82% (95% CI: 3.75%-8.32%, I^2^: 57.90%)	0%-4.76%
Dyspnea	46.06% (95% CI: 25.58%-66.54%, I^2^:95.3%)	1.47% (95% CI: 0.44%-2.50%, I^2^:14.2%)	0-0.2%
Cough	40.09% (95% CI: 20.38%-59.81%, I^2^:94.6%)	0%-15%	0%
Hemoptysis	0.2%-5%	0%-0.2%	0%-0.2%
Pleural effusion	7.5%	0%	0%
Lung infection	25%	20%	0%
Pulmonary embolism	2.7%	2.7%	0%
Emphysema	0.21%	0.21%	0.21%
Respiratory failure	1.36% (95% CI: 0.00%-4.35%, I^2^:51.9%)	1.36% (95% CI: 0.00%-4.35%, I^2^:51.9%)	0.2% (95% CI: 0.00%-0.7%, I^2^:0.0%)
Cardiac			
Acute coronary syndrome	5%	2.5%	0%
Sinus tachycardia	2.7%	2.7%	0%
Atrial fibrillation	2.7%-5%	0%-2.7%	0%
Myocardial infarction	0.21%-2.56%	0.21%-2.56%	0.21%-2.56%
Heart failure	2.5%	2.5%	0%
Cardiac arrest	0.42%	0.42%	0.42%
Cardiomyopathy	0.21%	0.21%	0.21%
Cardiopulmonary failure	0.21%	0.21%	0.21%
Myocarditis	0.76%	0.76%	0%
Aortic dissection	0.21%	0.21%	0.21%
Pericarditis	0.25%	0.25%	0%
Right ventricular failure	0.21%	0.21%	0.21%
Gastrointestinal			
Dysphagia	10%-82.5%	0%	0%
Esophagitis	38.51% (95% CI: 6.79%-70.22%, I^2^:98.5%)	0%-2.7%	0%
Anorexia	23.86% (95% CI: 3.02%-44.69%, I^2^:86.1%)	0%	0%
Nausea	30.51% (95% CI: 4.38%-56.64%, I^2^:92.0%)	0%-5%	0%
Vomiting	5%	0%	0%
Weight loss	20%	0%	0%
Constipation	27.67% (95% CI: 0.0%-58.04%, I^2^:96.7%)	0%-2.7%	0%
Diarrhea	17.30% (95% CI: 13.36%-21.23%, I^2^:39.0%)	0.6% (95% CI: 0.0%-1.32%, I^2^:0%)	0%
Gastric hemorrhage	2.5%-8.1%	2.5%-8.1%	0%-2.7%
Elevated liver enzymes	7.64% (95% CI: 4.65%-11.3%, I^2^:0.0%)	7.64% (95% CI: 4.65%-11.3%, I^2^:0.0%)	0%
Dehydration	5%-12.5%	0%-7.5%	0%
Colitis	4.24% (95% CI: 1.15%-7.34%, I^2^:0.0%)	1.52% (95% CI: 0.0%-3.80%, I^2^:0.0%)	0%
Endocrine			
Hyperthyroid	10%-10.8%	0%	0%
Hypothyroidism	10.73% (95% CI: 5.95%-15.51%, I^2^:57.8%)	0.25% (95% CI: 0.0%-0.74%, I^2^:0.0%)	0%
Adrenal insufficiency	5.4%	5.4%	0%
Hematologic			
Anaemia	5%-47.6%	2.5%-4.76%	0%
Febrile neutropenia	2.7%-5%	2.7%-5%	0%
Neutropenia	12.5%-38.1%	2.5%-9.5%	0%
Leucopenia	47.6%	4.7%	0%
Thromboembolism	5%	2.5%	0%
Hypercalcemia	2.5%-5%	0%-2.5%	0%
Other			
Fatigue	50.97% (95% CI: 25.61%-76.33%, I^2^:97.7%)	0%-5%	0%
Alopecia	35%	0%	0%
Paresthesia-	27.5%	0%	0%
Fever	32.5%-38.1%	0%	0%
Back pain	10.5%-17.5%	0%-0.21%	0%
Pruritus	11.58% (95% CI: 7.94%-15.23%, I^2^:23.8%)	0% (95% CI: 0.0%-0.29%, I^2^:0.0%)	0%
Rash	12.40% (95% CI: 6.47%-18.33%, I^2^:60.0%)	0%-5%	0%
Hypertension	17.5%	0%	0%
Hypotension	2.5%-5.0%	2.5%-5.0%	0%
Dizziness	15%	0%	0%
Asthenia	10.73%	0.63%	0%
Dermatitis	16.64% (95% CI: 3.87%-29.41%, I^2^:88.2%)	1.32% (95% CI: 0.0%-2.91%, I^2^:0.0%)	0%
Arthralgia	8.34% (95% CI: 1.21%-15.47%, I^2^:85.1%)	0%-2.7%	0%
Kidney injury	7.73% (95% CI: 1.35%-18.70%, I^2^:83.4%)	0.1% (95% CI: 0.0%-1.08%, I^2^:10.4%)	0%
Edema	7.5%-12.5%	0%	0%
Myalgia	5%-17.4%	0%-0.7%	0%
Headache	3.4%-10.9%	0%-5.4%	0%
Flu-like Illness	1.48%-5%	0%	0%
Infection	8.23% (95% CI: 1.59%-14.87%, I^2^:82.7%)	0.2%-20%	0%
Malaise	2.4%-10%	0%	0%
Hypothermia	2.7%	2.7%	0%
Myositis	5%	0%-5%	0%
Arthritis	2.17%	0%	0%
Chills	5%	0%	0%
Dysgeusia	5.0%	0%	0%
Peripheral neuropathy	45%	12.5%	0%
Seizure	2.7%	2.7%	0%
Sepsis	0.2%-2.5%	0.2%-2.5%	0%-0.2%
Thromboembolism	1.91% (95% CI: 0.25%-5.07%, I^2^:25.0%)	0.27%-2.5%	0%

ICIs, Immune checkpoint inhibitors; RT, Radiotherapy.

### 3.3 Pneumonitis

**Figure 4:**
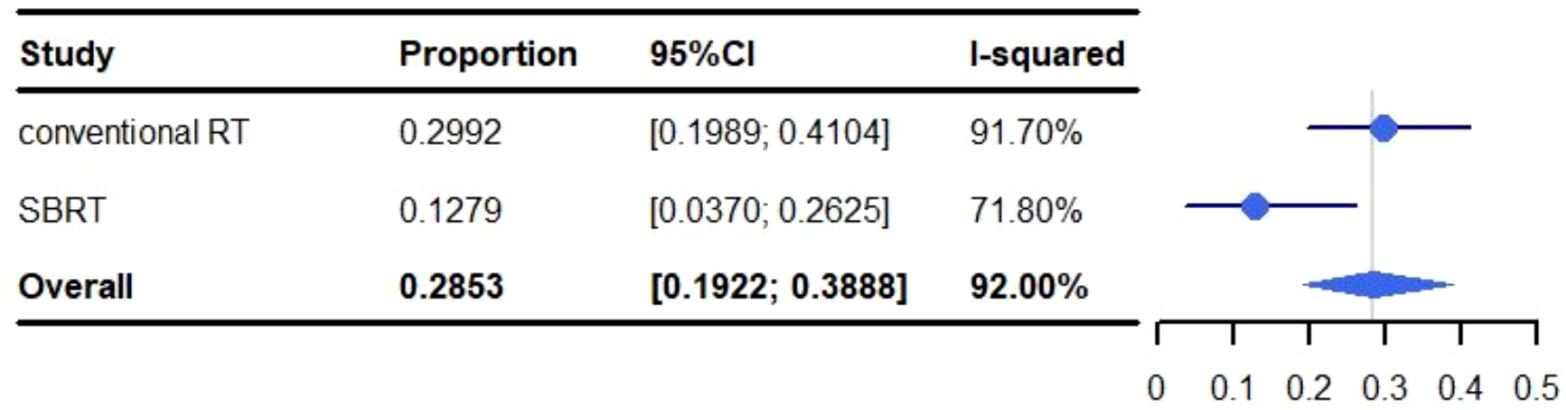
Meta-analysis and forest plots for non-small cell lung cancer patients treated with radiotherapy/chemoradiotherapy and immune checkpoint inhibitors who experienced grade 1-5 pneumonitis.

The pooled rate of grade 1-5 pneumonitis was 28.53% (95% CI: 19.22%-38.88%, I^2^: 92.00%). For conventional RT, the incidence of grade 1-5 pneumonitis was 29.92% (95% CI: 19.89%-41.04%, I^2^: 91.70%), and 12.79% (95% CI: 3.70%-26.25%, I^2^: 71.80%) for SBRT (**Figure 4**).

**Figure 5:**
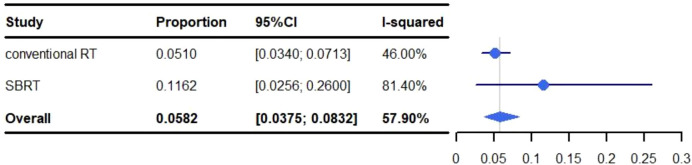
Meta-analysis and forest plots for non-small cell lung cancer patients treated with radiotherapy/chemoradiotherapy and immune checkpoint inhibitors who experienced grade 3-5 pneumonitis.

The pooled rate of grade 3-5 pneumonitis was 5.82% (95% CI: 3.75%-8.32%, I^2^: 57.90%). For conventional RT the occurrence of grade 3-5 pneumonitis was 5.10% (95% CI: 3.40%-7.13%, I^2^: 46.00%), and 11.62% (95% CI: 2.56%-26.00%, I^2^: 81.40%) for SBRT (**Figure 5**).

The statistical analysis for grade 5 pneumonitis analysis was not reported in this study due to the detection of publication bias. Nevertheless, the rate of grade 5 pneumonitis was 0%-4.76% overall, with 0%-4.76% for conventional RT and 0% for SBRT.

#### 3.3.1 Analyzing the relationship between radiotherapy dose and the incidence of pneumonitis

**Figure 6:**
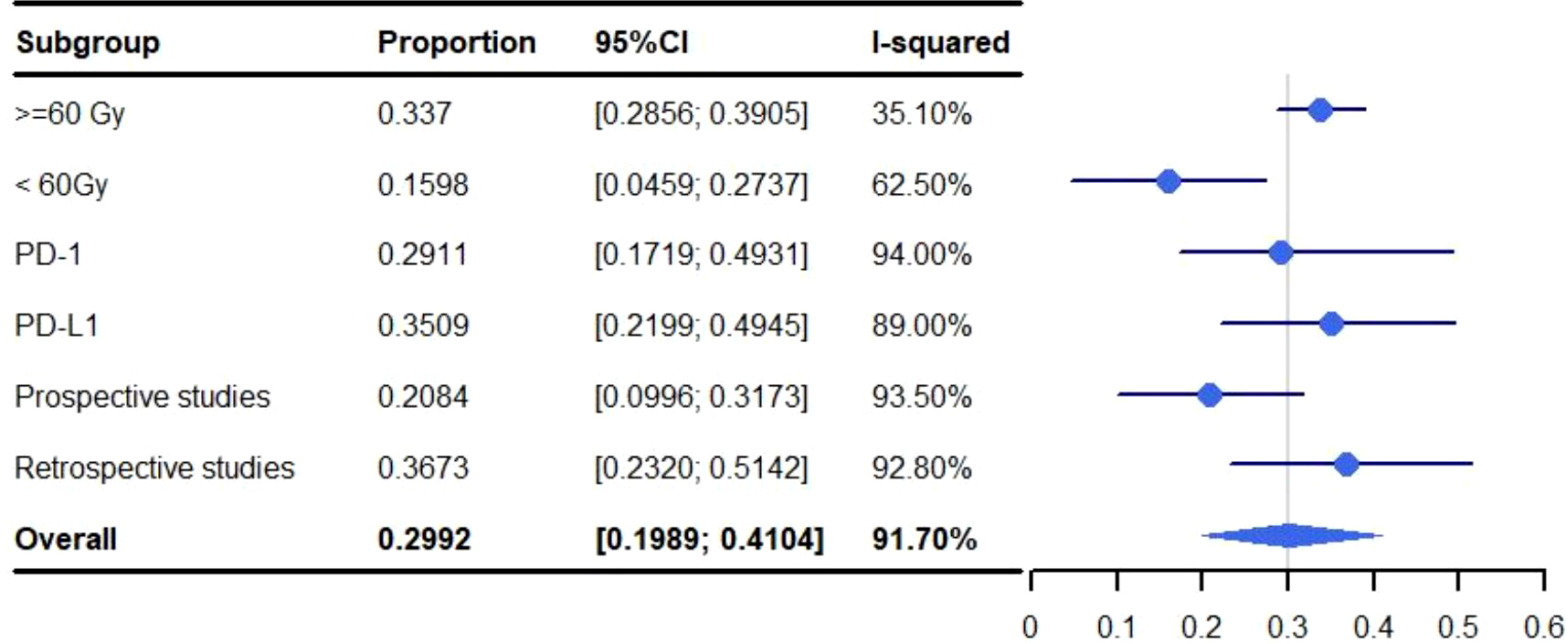
Subgroup analysis of meta-analysis and forest plot for non-small cell lung cancer patients treated with radiotherapy/chemoradiotherapy and immune checkpoint inhibitors who experienced grade 1-5 pneumonitis.

Grade 1-5 pneumonitis was higher in studies that used a total radiation dose >=60 Gy when compared to those that limited the total radiation dose to <60 Gy. With a grade 1-5 rate for pneumonitis of 33.70% versus 15.98% (>=60 vs. <60: 95%, CI: 28.56%-39.05%, I^2^: 35.1% and 95% CI: 4.59%-27.37%, I^2^: 62.50%, respectively) (**Figure 6**). A subgroup analysis was not performed for SBRT alone because only two articles were available.

In our study, only one trial (42) explored the relationship between tumor location and pneumonitis; there was no increased risk of pneumonitis in the lower lobe when compared to the middle/upper lobe. Moreover, only one trial explored the effects of different radiotherapy techniques (combined with ICIs) on pneumonitis (51); for which no difference was identified, perhaps due to the low number of patients included.

#### 3.3.2 Analyzing the relationship between immunological agents/duration of ICIs and the incidence of pneumonitis

**Figure 7:**
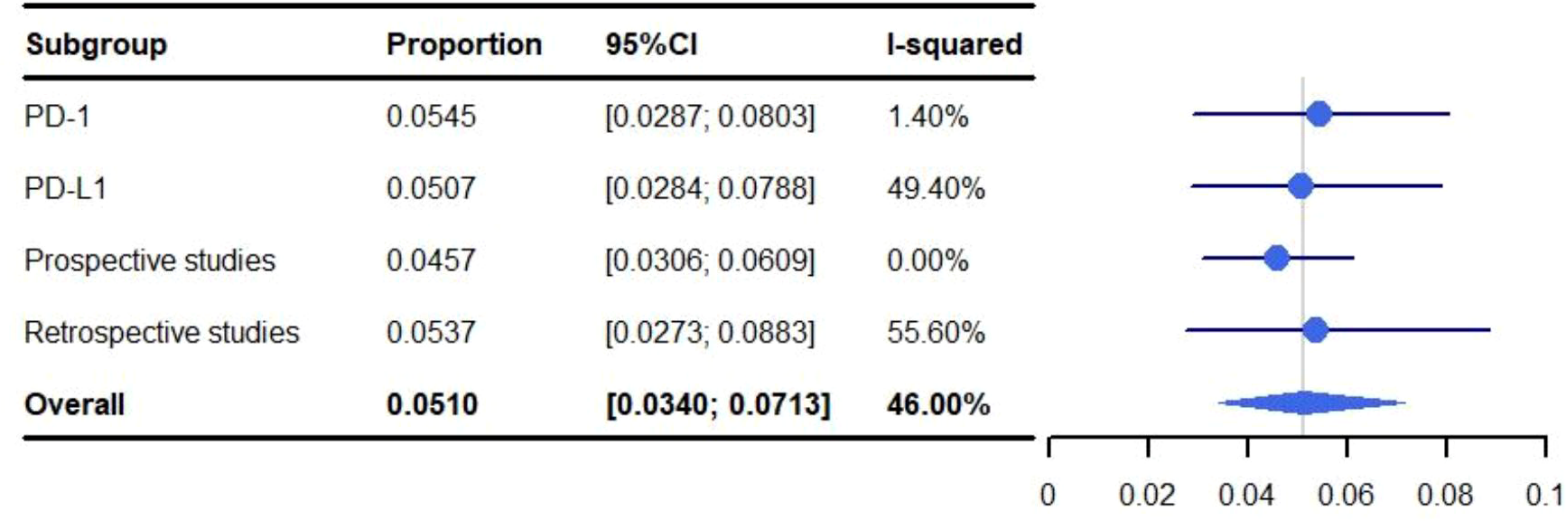
Subgroup analysis of meta-analysis and forest plot for non-small cell lung cancer patients treated with radiotherapy/chemoradiotherapy and immune checkpoint inhibitors who experienced grade 3-5 pneumonitis.

The pooled rate of grade 1-5 pneumonitis for patients treated with ICIs programmed cell death-ligand 1 (PD-L1) was 35.09% (95% CI: 21.99%-49.45%, I^2^: 89.0%) which was higher than those targeting programmed cell death protein-1 (PD-1) (29.11%; 95% CI: 17.19%-49.31%, I^2^: 94.00%). The rate of grade 3-5 pneumonitis for PD-L1 was 5.07% (95% CI: 2.84%-7.88%, I^2^: 49.4%) and 5.45% (95% CI: 2.87%-8.03%, I^2^: 1.4%) for PD-1 (**Figures 6**, **7**). A subgroup analysis was not performed for SBRT alone because only two articles were available.

When ICIs were administered for more than one year, the rate of grade 1-5 and grade 3-5 pneumonitis was 26.57% (95% CI: 15.70%-37.44%, I^2^: 89.1%) and 4.79% (95% CI: 3.26%-6.32%, I^2^: 0.6%), respectively. The rate of grade 1-5 and 3-5 pneumonitis for patients administered ICIs for less than one year, was 0%-17.24% and 0%-6.90%, respectively.

#### 3.3.3 Analyzing the relationship between prospective/retrospective research and the incidence of pneumonitis

Pneumonitis was identified at a higher rate in retrospective studies than prospective studies at both grade 1-5 (36.73% vs. 20.84%; 95% CI: 23.20%-51.42%, I^2^: 92.80 and 95% CI: 9.96%-31.73%, I^2^: 93.50%) and grade 3-5 (5.37% vs. 4.57%; 95% CI: 2.73%-8.83%, I^2^: 55.6% and 95% CI: 3.06%-6.09%, I^2^: 0.0%) (**Figures 6**, **7**). A subgroup analysis was not performed for SBRT alone because only two articles were available.

### 3.4 Publication bias

The Begg’s and Egger’s tests are enumerated in **Supplementary Materials Table 4**.

## 4 Discussion

The study included herein provides an overview of published NSCLC trials focusing on the use of RT with ICIs. This review and meta-analysis systematically, quantitatively and comprehensively analyzed the efficacy and toxicity of RT when combined with ICIs for 1,645 NSCLC patients from 25 studies.

The efficacy of RT when combined with ICIs (ORR, 45-45.9%) appeared to be superior for patients that had previously received treatment for metastatic NSCLC. The efficacy (ORR) for nivolumab or pembrolizumab against pretreated metastatic NSCLC ranged from 19 to 36% in the Checkmate 017, Checkmate 057 and Keynote 011 trials (52, 53). The one-year OS for metastatic NSCLC patients was 50% and the two-year OS was 25%, which was consistent with the aforementioned studies (one-year OS:42%-58%, two-year OS:23%-29%).

While concurrent CRT has been the standard treatment for inoperable cancer; durvalumab has been widely used as maintenance therapy following CRT, which was based upon evidence from the PACIFIC study (30). In our study, the pooled 1-year PFS was 56.39% (95% CI: 50.66%-62.03%, I^2^:39.4%), one-year OS was 83.25% (95% CI: 79.42%-86.75%, I^2^:17.6%) and two-year OS was 66.16% (95% CI: 62.30%-69.92%, I^2^:0.0%). When viewed in relation to other recent reports, the one-year PFS was 48% in both the 60 Gy group from RTOG 0617 (54) and the cisplatin-pemetrexed group from PROCLAIM (55), and 55.9% in the durvalumab group of PACIFIC. The one-year OS for RTOG 0617, PROCLAIM and PACIFIC was 78%, 76%, 83.1%, respectively. Therefore, RT/CRT when combined with ICIs may improve OS, PFS, and tumor response rates in NSCLC patients, although further study may be needed.

Zhou et al. (56), found that the overall incidence of grade 3-5 adverse events (AEs) ranged from 35% to 40% in PD-1 or PD-L1 monotherapy arms, while a rate of 40% to 50% AEs was observed when ICIs were combined with CRT (for 23,322 patients from 52 randomized controlled trials). In our study, the pooled rate of grade 3-5 AEs was 30.18% (95% CI: 10.04%-50.33%, I^2^: 96.7%) and 2.03% (95% CI: 0.03%-4.04%, I^2^: 36.8%) for grade 5 AEs, which was lower than the aforementioned study.

Cardiotoxicity is a rare but potentially fatal adverse effect of ICI therapy, for NSCLC patients, the overall incidence of cardiotoxicity is low (0%-5%); but the mortality associated with cardiotoxicity is high (0%-2.56%). We found that the most common form of lethal cardiotoxicity was myocardial infarction (0.21%-2.56%). In our study, the incidence of grade 1-5 myocarditis was 0.76%, which is within the range found by previous studies (0.05%-1.14%) (57, 58). RT&ICIs did not appear to increase the rate of myocarditis. However, Mahmood et al. (58), found that myocarditis (early during treatment) was more common following ICI treatment with ICI occurred, thus further research may be warranted.

Overall, the most common grade 5 adverse event was pneumonitis (0%-4.76%). Previous meta-analyses have demonstrated that the incidence of radiation pneumonitis is 1.5%–40%, with a 1.1%-6.6% incidence of grade ≥ 3 pneumonitis and 0%-3.1% for grade 5 (59, 60). In our study, the pooled incidence of pneumonitis was 28.53% (95% CI: 19.22%-38.88%, I^2^: 92.00%), with a 5.82% (95% CI: 3.75%-8.32%, I^2^: 57.90%) incidence of grade ≥ 3 pneumonitis and 0%-4.76% for grade 5. Thus, the incidence of pneumonitis following RT&ICIs is similar to RT alone, and likely safe for use in NSCLC patients.

There remain concerns among researchers in the field that the RT&ICI prospective studies may not reflect the real-world clinical application. For example, patients with a history of interstitial lung disease were excluded from prospective trials. This notion is supported by Suresh et al. (61), who found a higher incidence of immune-associated pneumonia in real-world setting. It is notable that we found that the incidence of grade 1-5 and grade 3-5 pneumonitis to be higher in retrospective studies than in the prospective studies (36.73% vs. 20.84% and 5.37% vs. 4.57%, respectively).

Only one trial (42) included in this study explored the relationship between tumor location and pneumonitis, and no increased risk of pneumonitis was observed in the lower lobe compared with the middle/upper lobe. However, further research is warranted since Zhang et al. (62) found that tumors located in the lower lobe were associated with a significantly increased probability of grade ≥2 radiation-pneumonitis.

Elective node irradiation is performed for NSCLC patients to treat lymph nodes that are not known to contain metastases, including the bilateral hilar, mediastinum, and even supraclavicular areas. Grills et al. (63), has suggested that elective node irradiation would increase the volume of the radiotherapy target, lead to toxicity and make it hard to improve the therapeutic dose. The administration of immunotherapy further complicates the choice between involved-field and elective node irradiation. Previous studies have demonstrated that direct irradiation of lymph nodes can partially explain lymphopenia (64, 65). Additionally, there is increasing evidence that lymphocyte depletion in NSCLC is associated with a poorer prognosis, due to the lower likelihood of a robust immunotherapeutic response (66). It is possible that involved-field irradiation may have greater synergy with ICIs, and form a more a powerful RT strategy in the era of immunotherapy.

In addition to involved-field irradiation, clinical target volume (CTV) omission is another strategy that has been used in conjunction with immunotherapy. CTV is defined as the volume of tissue containing gross tumor volume and subclinical microscopic malignant lesions. A retrospective study of 105 stage III NSCLC patients treated with (n=50) or without (n=55) CTV did not experience a significantly different rate of local recurrence, distant metastasis, PFS or OS. Notably, the incidence of grade 3–4 radiation-pneumonitis was significantly lower in group that did not receive CTV (P=0.044) (67). Kilburn et al. (68), found that only 1.8% of patients (2/110) experienced CTV (planning target volumes expanded 1 cm) failures, suggesting that CTV omission appears to be a viable strategy, further study may be warranted.

We found higher rates of grade 1-5 pneumonitis in studies where a total radiation dose of >=60 Gy (vs. < 60 Gy) was administered (33.70% vs. 15.98%). Prior to the advent ICIs, the RTOG 7301 trial established that the standard radiation dose for unresectable NSCLC was 60–63 Gy in 1.8–2 Gy daily fractions (69). Subsequently, the RTOG 0617 trial identified that dose escalation from 60 to 74 Gy resulted in worse OS when compared to 60 Gy (70). In addition to dose escalation, there has been considerable interest in the potential benefits of unconventional fractionated radiotherapy for NSCLC. A meta-analysis has shown that hyper-fractionated and accelerated radiotherapy can yield a modest survival benefit (compared to conventional regimens) for NSCLC patients (71).

A number of immune system effects have been associated with RT, for example Crittenden et al. (72) demonstrated that immunosuppressive tumor-associated macrophages occur in the presence of high-dose irradiation (60 Gy). A significant reduction in myeloid-derived suppressor cells has also been observed 7–14 days after single high-dose RT for colonic tumors in mice (73). Other studies also found that single dose of 8 to 10 Gy is more immunogenic than a traditional grading protocol (74–76). Therefore, hypo-fractionated radiation therapy (HFRT) has been suggested as being more likely to activate immune effects. A pooled analysis of the PEMBRO-RT and MDACC trials found that ablative radiotherapy combined with pembrolizumab had a significantly (*P* < 0.05) better ORR (48% and 54% for 24Gy/3 and 50Gy/4, respectively) than both non-ablative radiotherapy (18% ORR with 45Gy/15 fractions) or pembrolizumab (20% ORR) alone (77). Additionally, low-dose radiation has been suggested as a means of producing a systemic immune effect (78). Several studies have explored the use of low-dose radiotherapy (LDRT) (0.5 to 2.0 Gy, with 1 or a few fractions) to enhance the abscopal response of distant tumors and improve the immunogenicity of “cold tumors” (79, 80). Limei Yin et al. (81) demonstrated that patients with stage IV NSCLC have a better systemic antitumor with a triple treatment consisting of LDRT, HFRT and ICIs, partial response was achieved for three patients and stable disease for two patients. Taken together, these findings suggest that different dose-fractionation regimens may have different effects on the immune system and dose-fractionation schedules should be re-evaluated to determine the optimal RT&ICI regimen.

Radiotherapy dose distribution also plays an important role, according to results from the phase III RTOG 0617 clinical trial, a V20 dose of less than 35% can minimize the risk of pneumonitis (70). Shaikh et al. (82) suggested that a V5 of <65% and V20 of <25% reduced the risk of grade 2 radiation-pneumonitis. Additionally, the dose constraints were tightened when employing ICIs in combination with CRT. Landman et al. (32) found that a V5 of <55%, V20 <23% and mean lung dose <14.8 Gy were the thresholds for pneumonitis when combined with ICIs. Jang et al. (42) identified that a mean lung dose of 16 Gy, V30 and V40 could significantly predict the occurrence of radiation-pneumonitis following ICI treatment; and in the non-ICI group, only V20 could significantly predict occurrence of radiation-pneumonitis. They also found that V40 had the largest area under the curve among various parameters in the ICI group. Hassanzadeh et al (34), found that pneumonitis-free survival rate was correlated with lung V5; while lung V20 and mean lung dose were not significantly correlated with pneumonitis. Schoenfeld et al. (83) also suggested that low dose radiation exposure (represented by V5) may be more important in pneumonitis risk when consolidation ICIs are used. Further studies of dosimetric parameters and their association with pneumonitis are warranted.

Our study identified only one trial that investigated the effects on pneumonitis for different radiotherapy techniques in combination with ICIs (51). However, few patients were included and no differences were found. Advances in radiotherapy technology could be particularly important when paired with ICI therapy. The shift from two-dimensional radiotherapy to advanced three-dimensional-based radiation techniques, including 3-dimensional conformal radiotherapy and intensity-modulated radiotherapy, allows for more precise radiation delivery and reduced exposure to adjacent critical structures. Recently, intensity-modulated radiotherapy was found to provide similar outcomes (OS and PFS) with a significantly lower risk of radiation pneumonitis when compared to 3-dimensional conformal radiotherapy (3.5% versus 7.9%, respectively) (84). There are few studies on the application of new radiotherapy techniques with ICIs.

An analysis of two prospective metastatic NSCLC trials reported that the PFS associated with SBRT when combined with anti-PD1 treatment was significantly better than anti-CTLA4; although the efficacy was not statistically different (11). Gu et al. (85) found an increased likelihood of hypothyroidism after PD-1 inhibition against lung cancer, and renal injury for PD-L1. Pneumonitis was more common following PD-1 treatment, but hepatitis, rash and lipase elevation were more common for PD-L1. In our study, we found grade 3-5 pneumonitis was higher for patients treated with PD-1 inhibitors than PD-L1 inhibitors (5.45% vs. 5.07%), which is consistent with other studies (56, 85–87). Inhibition of PD-1 and blockage of PD-1-PD-L2 may contribute to cytokine release and proliferation of autoreactive T cells, leading to an enhanced antitumor effect and AEs (88). We also found that grade 1-5 pneumonitis was lower for PD-1 inhibitors than PD-L1 inhibitors (29.11% vs. 35.09%). However, many retrospective studies focused upon the use of PD-L1 in our study and further research is necessary. Li et al. (87) suggested that RT may be the leading factor for these differences, rather than the ICIs themselves. This would account for the similar incidence of AEs following treatment using PD-1 and PD-L1 inhibitors when combined with RT, which indicates that selection of ICIs should be mainly based upon their efficacy rather than toxicity.

We also found that the administration of ICIs for more than one year was associated with a 26.57% (95% CI: 15.70%-37.44%, I^2^: 89.1%) rate of grade 1-5 pneumonitis and a 4.79% (95% CI: 3.26%-6.32%, I^2^: 0.6%) rate of grade 3-5 pneumonitis. When the ICI treatment lasted for less than one year, the rate of grade 1-5 and grade 3-5 pneumonitis was 0%-17.24% and 0%-6.90%, respectively. Sukari et al. (89) found the duration of PD1 was significantly associated with the development of pneumonitis, and Shankar et al. (90) also found that longer ICI treatment was associated with a higher incidence of immune-related AEs (and a greater therapeutic effect). Therefore, the increased potential for AEs should be considered when prolonged extended treatment regimens.

No definitive conclusions can be made regarding the optimal schedule for the administration of RT/CRT with ICIs. A retrospective analysis from a prospective study showed patients treated with pembrolizumab (and prior RT) showed improved PFS and OS (14); another study reached the opposite conclusion, that prior RT was associated with poorer survival (91). One study found that metastatic cancer (80% lung cancer and 20% other cancers) patients who received (SBRT/SRT) after completion of immunotherapy (3.6 months) had significantly worse OS (P=0.01) than those who received SRT prior to or concurrent with immunotherapy (13.0 months) (92). However, Lesueur et al. suggested that there may be no OS or PFS differences for metastatic NSCLC patients treated with RT before or during/after ICI administration (93). Interestingly, some preclinical studies have suggested that the optimal timing may depend on the type of ICI. Anti-CTLA-4 therapies were found to be most effective when administered before RT; whereas the optimal timing of anti-OX40 delivery was one day following RT, during the increased antigen presentation post-radiation window (94).

The PACIFIC trial (95) found that consolidative durvalumab reduced the risk of progression more effectively and improved the survival rate when initiated following concurrent CRT within two weeks or less (PFS: hazard ratio (HR), 0.42 [CI, 0.29–0.61], OS: HR, 0.53 [CI, 0.35–0.79]), compared to more than two weeks (PFS: HR, 0.65 [CI, 0.52–0.81], OS: HR, 0.78 [CI, 0.61–0.99]). Bassanelli et al. (96) suggested that the first ≤60 days prior to the completion of RT (mOS, 22.4 months vs. 8.6 months, p = 0.005) was the most appropriate window for ICI administration. However, Bryant et al. (97) found that initiation of durvalumab ≤14 days after RT was not associated with improvement in PFS or OS. Denault et al. (98) also found no significant difference in efficacy or toxicity between two and four weeks durvalumab therapy for patients treated with CRT. Recently, Anscher et al. (99) identified that RT administered within 90 days prior to ICI did not increase the risk of serious AEs using data for 16,835 patients from 68 prospective trials. Patients who were administered ICIs and RT within ≤90 days had slightly higher rates of fatigue, endocrine disorders and pneumonitis than those without RT. These differences were due to low-grade (grade 1-2) AEs, thus ICI administration within 90 days of RT appears to be safe.

Some difficulties remain for the identification of patients that will gain the greatest survival benefit from combination therapy or those who are more likely to have adverse effects. PD-L1 and tumor mutation burden (TMB) were widely used in clinical stratification of ICI patients, but their predictive effects are still controversial. Many other markers based upon tumor tissue, peripheral blood, and radiological images have been investigated, including: cell surface markers, various immune cells, immune related gene and imaging biomarkers (100). However, they showed limited ability to distinguish non-responders from responders following RT combined with ICI therapy.

It is important to note that most of the studies included in this meta-analysis were single-arm clinical trials, which prevents the comparison of the advantages and disadvantages associated with CRT/RT + ICIs and CRT/RT based upon a balanced baseline. Whilst the results from these trials may contain a high amount of statistical heterogeneity, subgroup analyses were performed to identify sources of study heterogeneity. Finally, due to data availability for lung cancer patients in this field, we could not explore certain details regarding the efficacy and safety of RT/CRT plus ICIs, including: the influence of different radiotherapy doses/fractionations, irradiation target volumes, chemotherapy regimen.

## 5 Conclusion

In conclusion, this comprehensive meta-analysis of 1,645 non-small cell lung cancer patients (from 25 studies) systemically and quantitatively explored the clinical efficacy and safety of immune checkpoint inhibitors when combined with radiotherapy/chemoradiotherapy. Based upon the studies presented herein, the combination of (chemo)radiotherapy and immune checkpoint inhibitors appears to be a both safe and feasible mode of treatment. These findings may help clinicians in the design of future trials testing concurrent or sequential combinations of immune checkpoint inhibitors and radiotherapy/chemoradiotherapy for the treatment of patients with non-small cell lung cancer.

## Data availability statement

The original contributions presented in the study are included in the article/**Supplementary Material.** Further inquiries can be directed to the corresponding authors.

## Author contributions

YuZ and YaZ conceptualized the study. JW, RD, YL, CF and TN collected the data. JW, TN, QinZ, FT, QiZ and YX analyzed the data. JW and YZ wrote the manuscript. All authors contributed to the article and approved the submitted version.
