# Supplementary material for: Safety and efficacy of radiotherapy/chemoradiotherapy combined with immune checkpoint inhibitors for non-small cell lung cancer: A systematic review and meta-analysis
Source: Front Immunol. 2023 Mar 13;14:1065510. doi: 10.3389/fimmu.2023.1065510 (PMC10040597; doi:10.3389/fimmu.2023.1065510)
Supplement: Supplementary file 1 [file Table_1.docx]

**Supplemental Table 1** Search Strategy

| Database |  |
| --- | --- |
| Pubmed | ((("non-small cell lung neoplasm (s) "[Title/Abstract]) OR ("non-small cell lung cancer (s) "[Title/Abstract]) OR ("non-small cell lung carcinoma(s) "[Title/Abstract]) OR ("NSCLC "[Title/Abstract]))) AND ((("radiotherapy"[All Fields]) OR ("radiation therapy "[All Fields]) OR ("radiation treatment"[All Fields]) OR ("irradiation"[All Fields]) OR ("SABR"[All Fields]) OR ("stereotactic ablative radiotherapy"[All Fields]) OR ("SBRT"[All Fields]) OR ("stereotactic body radiation therapy"[All Fields]) OR ("SRS"[All Fields]) OR ("stereotactic radiosurgery"[All Fields]) OR ("SRT"[All Fields]) OR ("stereotactic radiotherapy"[All Fields]) OR ("radio-chemotherapy"[All Fields]) OR ("chemoradiotherapy"[All Fields]))) AND ((("immunotherapy"[All Fields]) OR ("immune checkpoint inhibitors"[All Fields]) OR ("checkpoint inhibitor"[All Fields]) OR ("checkpoint blockade"[All Fields]) OR ("programmed cell death 1 receptor"[All Fields]) OR ("programmed cell death 1 ligand 1"[All Fields]) OR ("programmed death-1"[All Fields]) OR ("PD-1"[All Fields]) OR ("programmed death ligand-1"[All Fields]) OR ("PD-L1"[All Fields]) OR ("cytotoxic T lymphocyte-associated antigen-4 (CTLA-4) antigen"[All Fields]) OR ("anti-CTLA-4"[All Fields]) OR ("anti-PD-1"[All Fields]) OR ("anti-PD-L1"[All Fields]) OR ("CTLA-4"[All Fields]) OR ("Durvalumab"[All Fields]) OR ("Imfinzi"[All Fields]) OR ("MEDI-4736"[All Fields]) OR ("Atezolizumab"[All Fields]) OR ("MPDL3280A"[All Fields]) OR ("Tecentriq"[All Fields]) OR ("RO5541267"[All Fields]) OR ("RG7446"[All Fields]) OR ("Pembrolizumab"[All Fields]) OR ("Lambrolizumab"[All Fields]) OR ("Keytruda"[All Fields]) OR ("SCH 900475"[All Fields]) OR ("MK-3475"[All Fields]) OR ("Nivolumab"[All Fields]) OR ("Opdivo"[All Fields]) OR ("Ono-4538"[All Fields]) OR ("MDX-1106"[All Fields]) OR ("BMS-936558"[All Fields]) OR ("Nivo"[All Fields]) OR ("Avelumab"[All Fields]) OR ("Barvencik"[All Fields]) OR ("MSB0010718C"[All Fields]) OR ("Toripalima"[All Fields]) OR ("Tislelizumab"[All Fields]) OR ("Camrelizumab"[All Fields]) OR ("Sintilimab"[All Fields]) OR ("Tremelimumab"[All Fields]) OR ("Ipilimumab"[All Fields]) OR ("Cemiplimab"[All Fields]) OR ("Libtayo"[All Fields]) OR ("PDCD1"[All Fields]) OR ("CD274"[All Fields]))) |
| Web of Science | (TI=(("non-small cell lung neoplasm (s)”) OR ("non-small cell lung cancer (s)”) OR ("non-small cell lung carcinoma (s)”) OR (" NSCLC ”) OR AB=(("non-small cell lung neoplasm (s)”) OR ("non-small cell lung cancer (s)”) OR ("non-small cell lung carcinoma (s)”) OR (" NSCLC ”))) AND (TI=(("radiotherapy") OR ("radiation therapy ") OR ("radiation treatment") OR ("irradiation") OR ("SABR") OR ("stereotactic ablative radiotherapy") OR ("SBRT") OR ("stereotactic body radiation therapy") OR ("SRS") OR ("stereotactic radiosurgery") OR ("SRT") OR ("stereotactic radiotherapy") OR ("radio-chemotherapy") OR ("chemoradiotherapy") OR AB=(("radiotherapy") OR ("radiation therapy ") OR ("radiation treatment") OR ("irradiation") OR ("SABR") OR ("stereotactic ablative radiotherapy") OR ("SBRT") OR ("stereotactic body radiation therapy") OR ("SRS") OR ("stereotactic radiosurgery") OR ("SRT") OR ("stereotactic radiotherapy") OR ("radio-chemotherapy") OR ("chemoradiotherapy"))) AND (TI=(("immunotherapy") OR ("immune checkpoint inhibitors") OR ("checkpoint inhibitor") OR ("checkpoint blockade") OR ("programmed cell death 1 receptor") OR ("programmed cell death 1 ligand 1") OR ("programmed death-1") OR ("PD-1") OR ("programmed death ligand-1") OR ("PD-L1") OR ("cytotoxic T lymphocyte-associated antigen-4 (CTLA-4) antigen") OR ("anti-CTLA-4”) OR ("anti-PD-1") OR ("anti-PD-L1" OR ("CTLA-4") OR ("Durvalumab") OR ("Imfinzi") OR ("MEDI-4736") OR ("Atezolizumab") OR ("MPDL3280A") OR ("Tecentriq") OR ("RO5541267") OR ("RG7446") OR ("Pembrolizumab") OR ("Lambrolizumab") OR ("Keytruda") OR ("SCH 900475") OR ("MK-3475") OR ("Nivolumab") OR ("Opdivo") OR ("Ono-4538") OR ("MDX-1106") OR ("BMS-936558") OR ("Nivo") OR ("Avelumab") OR ("Barvencik") OR ("MSB0010718C") OR ("Toripalima") OR ("Tislelizumab") OR ("Camrelizumab") OR ("Sintilimab") OR ("Tremelimumab") OR ("Ipilimumab") OR ("Cemiplimab") OR ("Libtayo") OR ("PDCD1") OR ("CD274") OR AB=(("immunotherapy") OR ("immune checkpoint inhibitors") OR ("checkpoint inhibitor") OR ("checkpoint blockade") OR ("programmed cell death 1 receptor") OR ("programmed cell death 1 ligand 1") OR ("programmed death-1") OR ("PD-1") OR ("programmed death ligand-1") OR ("PD-L1") OR ("cytotoxic T lymphocyte-associated antigen-4 (CTLA-4) antigen") OR ("anti-CTLA-4”) OR ("anti-PD-1") OR ("anti-PD-L1" OR ("CTLA-4") OR ("Durvalumab") OR ("Imfinzi") OR ("MEDI-4736") OR ("Atezolizumab") OR ("MPDL3280A") OR ("Tecentriq") OR ("RO5541267") OR ("RG7446") OR ("Pembrolizumab") OR ("Lambrolizumab") OR ("Keytruda") OR ("SCH 900475") OR ("MK-3475") OR ("Nivolumab") OR ("Opdivo") OR ("Ono-4538") OR ("MDX-1106") OR ("BMS-936558") OR ("Nivo") OR ("Avelumab") OR ("Barvencik") OR ("MSB0010718C") OR ("Toripalima") OR ("Tislelizumab") OR ("Camrelizumab") OR ("Sintilimab") OR ("Tremelimumab") OR ("Ipilimumab") OR ("Cemiplimab") OR ("Libtayo") OR ("PDCD1") OR ("CD274"))) |
| Cochrane Library | (‘non-small cell lung neoplasm (s)’ : ti,ab OR ‘non-small cell lung cancer (s)’: ti,ab OR ‘non-small cell lung carcinoma (s)’ : ti,ab OR ‘ NSCLC ‘ : ti,ab) AND (‘radiotherapy’: ti,ab OR ‘radiation therapy’: ti,ab OR ‘radiation treatment’: ti,ab OR ‘irradiation’: ti,ab OR ‘SABR’: ti,ab OR ‘stereotactic ablative radiotherapy’: ti,ab OR ‘SBRT’: ti,ab OR ‘stereotactic body radiation therapy’: ti,ab OR ‘SRS’: ti,ab OR ‘stereotactic radiosurgery’: ti,ab OR ‘SRT’: ti,ab OR ‘stereotactic radiotherapy’: ti,ab OR ‘radio-chemotherapy’: ti,ab OR ‘chemoradiotherapy’: ti,ab ) AND (‘immunotherapy’: ti,ab OR ‘immune checkpoint inhibitors’: ti,ab OR ‘checkpoint inhibitor’: ti,ab OR ‘checkpoint blockade’: ti,ab OR ‘programmed cell death 1 receptor’: ti,ab OR ‘programmed cell death 1 ligand 1’: ti,ab OR ‘programmed death-1’: ti,ab OR ‘PD-1’: ti,ab OR ‘programmed death ligand-1’: ti,ab OR ‘PD-L1’: ti,ab OR ‘cytotoxic T lymphocyte-associated antigen-4 CTLA-4 antigen’: ti,ab OR ‘anti-CTLA-4’ : ti,ab OR ‘anti-PD-1’: ti,ab OR ‘anti-PD-L1’: ti,ab OR ‘CTLA-4’: ti,ab OR ‘Durvalumab’: ti,ab OR ‘Imfinzi’: ti,ab OR ‘MEDI-4736’: ti,ab OR ‘Atezolizumab’: ti,ab OR ‘MPDL3280A’: ti,ab OR ‘Tecentriq’: ti,ab OR ‘RO5541267’: ti,ab OR ‘RG7446’: ti,ab OR ‘Pembrolizumab’: ti,ab OR ‘Lambrolizumab’: ti,ab OR ‘Keytruda’: ti,ab OR ‘SCH 900475’: ti,ab OR ‘MK-3475’: ti,ab OR ‘Nivolumab’: ti,ab OR ‘Opdivo’: ti,ab OR ‘Ono-4538’: ti,ab OR ‘MDX-1106’: ti,ab OR ‘BMS-936558’: ti,ab OR ‘Nivo’: ti,ab OR ‘Avelumab’: ti,ab OR ‘Barvencik’: ti,ab OR ‘MSB0010718C’: ti,ab OR ‘Toripalima’: ti,ab OR ‘Tislelizumab’: ti,ab OR ‘Camrelizumab’: ti,ab OR ‘Sintilimab’: ti,ab OR ‘Tremelimumab’: ti,ab OR ‘Ipilimumab’: ti,ab OR ‘Cemiplimab’: ti,ab OR ‘Libtayo’: ti,ab OR ‘PDCD1’: ti,ab OR ‘CD274’: ti,ab) |

**Supplemental Table 2** Risk of Bias for non-randomized trials

| Study | D1 | D2 | D3 | D4 | D5 | D6 | D7 | Overall |
| --- | --- | --- | --- | --- | --- | --- | --- | --- |
| Shukla | Low | Low | Low | Low | Low | Low | Low | Low |
| Shaverdian | Low | Low | Low | Low | Moderate | Low | Low | Moderate |
| LeClair | Moderate | Low | Low | Low | Moderate | Low | Moderate | Moderate |
| Landman | Moderate | Low | Low | Low | Low | Low | Low | Moderate |
| Aredo | Moderate | Low | Low | Low | Moderate | Low | Low | Moderate |
| Hassanzadeh | Moderate | Low | Low | Low | Moderate | Low | Low | Moderate |
| Miura | Moderate | Low | Low | Low | Moderate | Low | Low | Moderate |
| Jung | Moderate | Low | Low | Low | Low | Low | Moderate | Moderate |
| Inoue | Moderate | Low | Low | Low | Low | Low | Moderate | Moderate |
| Chu | Moderate | Low | Low | Low | Moderate | Low | Moderate | Moderate |
| Lin | Low | Low | Low | Low | Low | Low | Low | Low |
| Yamaguchi | Moderate | Low | Low | Low | Moderate | Low | Moderate | Moderate |
| Tamiya | Moderate | Low | Low | Low | Moderate | Low | Low | Moderate |
| Jang | Moderate | Low | Moderate | Low | Low | Low | Low | Moderate |
| Barrón | Moderate | Low | Moderate | Low | Moderate | Low | Low | Moderate |
| Amino | Moderate | Low | Low | Low | Low | Moderate | Low | Moderate |
| Boyer | Moderate | Low | Moderate | Low | Low | Low | Low | Moderate |
| Bruni | Moderate | Low | Low | Low | Moderate | Low | Low | Moderate |
| Peters | Low | Low | Low | Low | Moderate | Low | Low | Moderate |
| Jabbou | Low | Low | Low | Low | Low | Moderate | Low | Moderate |
| Bestvina | Low | Low | Low | Low | Low | Low | Low | Low |
| Welsh | Low | Low | Low | Low | Low | Low | Moderate | Moderate |
| Chen | Moderate | Low | Low | Low | Low | Low | Low | Moderate |
| Voong | Moderate | Low | Low | Low | Low | Low | Moderate | Moderate |

**Supplemental Table 3** Risk of Bias for the randomized controlled trial

| Study | D1 | D2 | D3 | D4 | D5 | Overall |
| --- | --- | --- | --- | --- | --- | --- |
| Finn | Low | Moderate | Low | Low | Low | Moderate |

**Supplemental Table 4** Publication bias of the meta-analysis

| Subgroup | Begg’s test | Egger’s test |
| --- | --- | --- |
| 1-year PFS II-III stage NSCLC | 0.8806 | 0.7646 |
| 1-Year OS II-III stage NSCLC | 0.8510 | 0.8153 |
| 2-Year OS II-III stage NSCLC | 0.6015 | 0.9823 |
| Grade 3-5 adverse events | 0.3223 | 0.8494 |
| Grade 3-5 adverse events RT | 0.2931 | 0.9415 |
| Grade 5 adverse events | 0.1046 | 0.6351 |
| Grade 5 adverse events RT | 0.2582 | 0.4594 |
| Grade 1-5 Pneumonitis | 0.7969 | 0.8881 |
| Grade 1-5 Pneumonitis RT | 0.6909 | 0.8635 |
| Grade 1-5 Pneumonitis SBRT | 0.4969 | 0.6686 |
| Grade 3-5 Pneumonitis | 0.6112 | 0.4558 |
| Grade 3-5 Pneumonitis RT | 0.7202 | 0.7823 |
| Grade 3-5 Pneumonitis SBRT | 0.6015 | 0.3405 |
| Grade 1-5 Dyspnea | 0.4527 | 0.1383 |
| Grade 3-5 Dyspnea | 0.1765 | 0.2754 |
| Grade 1-5 Cough | 0.3223 | 0.7899 |
| Grade 1-5 Respiratory failure | 0.1172 | 0.1820 |
| Grade 3-5 Respiratory failure | 0.1172 | 0.1820 |
| Grade 5 Respiratory failure | 0.6015 | 0.1783 |
| Grade 1-5 Esophagitis | 0.2429 | 0.1970 |
| Grade 1-5 Anorexia | 0.6015 | 0.1767 |
| Grade 1-5 Nausea | 0.1885 | 0.2554 |
| Grade 1-5 Constipation | 0.3272 | 0.2874 |
| Grade 1-5 Diarrhea | 0.3476 | 0.9963 |
| Grade 3-5 Diarrhea | 0.5730 | 0.7544 |
| Grade 1-5 Elevated liver enzymes | 0.8429 | 0.1780 |
| Grade 3-5 Elevated liver enzymes | 0.8429 | 0.1780 |
| Grade 1-5 Colitis | 0.4969 | 0.1408 |
| Grade 3-5 Colitis | 0.1742 | 0.3005 |
| Grade 1-5 Hypothyroidism | 0.5730 | 0.7539 |
| Grade 3-5 Hypothyroidism | 0.4363 | 0.3284 |
| Grade 1-5 Fatigue | 0.4527 | 0.2145 |
| Grade 1-5 Pruritus | 0.8510 | 0.8891 |
| Grade 3-5 Pruritus | 0.1172 | 0.4876 |
| Grade 1-5 Rash | 0.2931 | 0.5975 |
| Grade 1-5 Dermatitis | 0.2429 | 0.1240 |
| Grade 3-5 Dermatitis | 0.2429 | 0.1609 |
| Grade 1-5 Arthralgia | 0.9999 | 0.8233 |
| Grade 1-5 Kidney injury | 0.2786 | 0.2658 |
| Grade 3-5 Kidney injury | 0.5389 | 0.7364 |
| Grade 1-5 Infection | 0.6242 | 0.8557 |
| Grade 1-5 Thromboembolism | 0.6015 | 0.2434 |
