# Supplementary figures and images for: Safety and efficacy of radiotherapy/chemoradiotherapy combined with immune checkpoint inhibitors for non-small cell lung cancer: A systematic review and meta-analysis
Source: Front Immunol. 2023 Mar 13;14:1065510. doi: 10.3389/fimmu.2023.1065510 (PMC10040597; doi:10.3389/fimmu.2023.1065510)

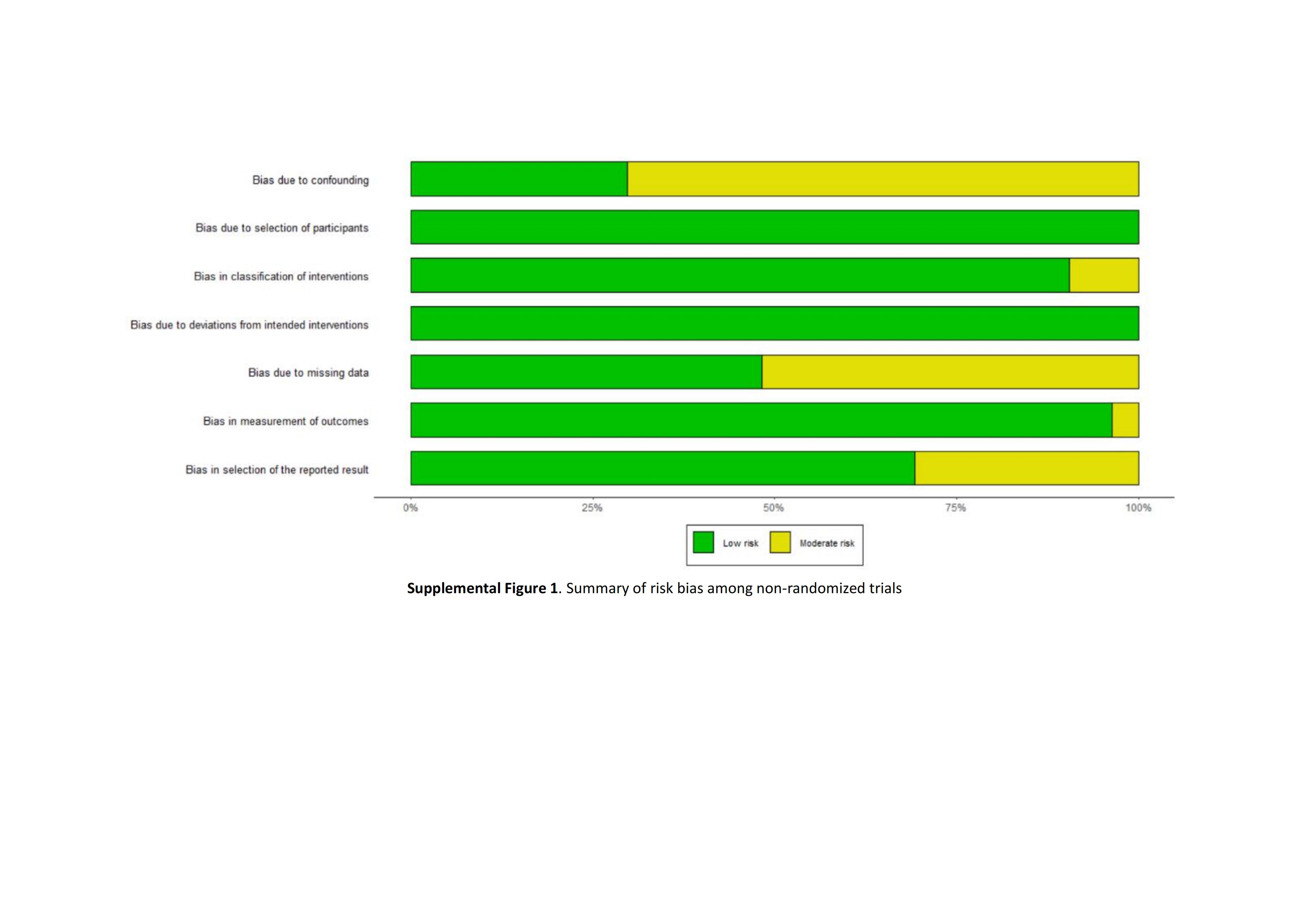

Supplement: Supplementary file 2 [file Image_1.jpeg]

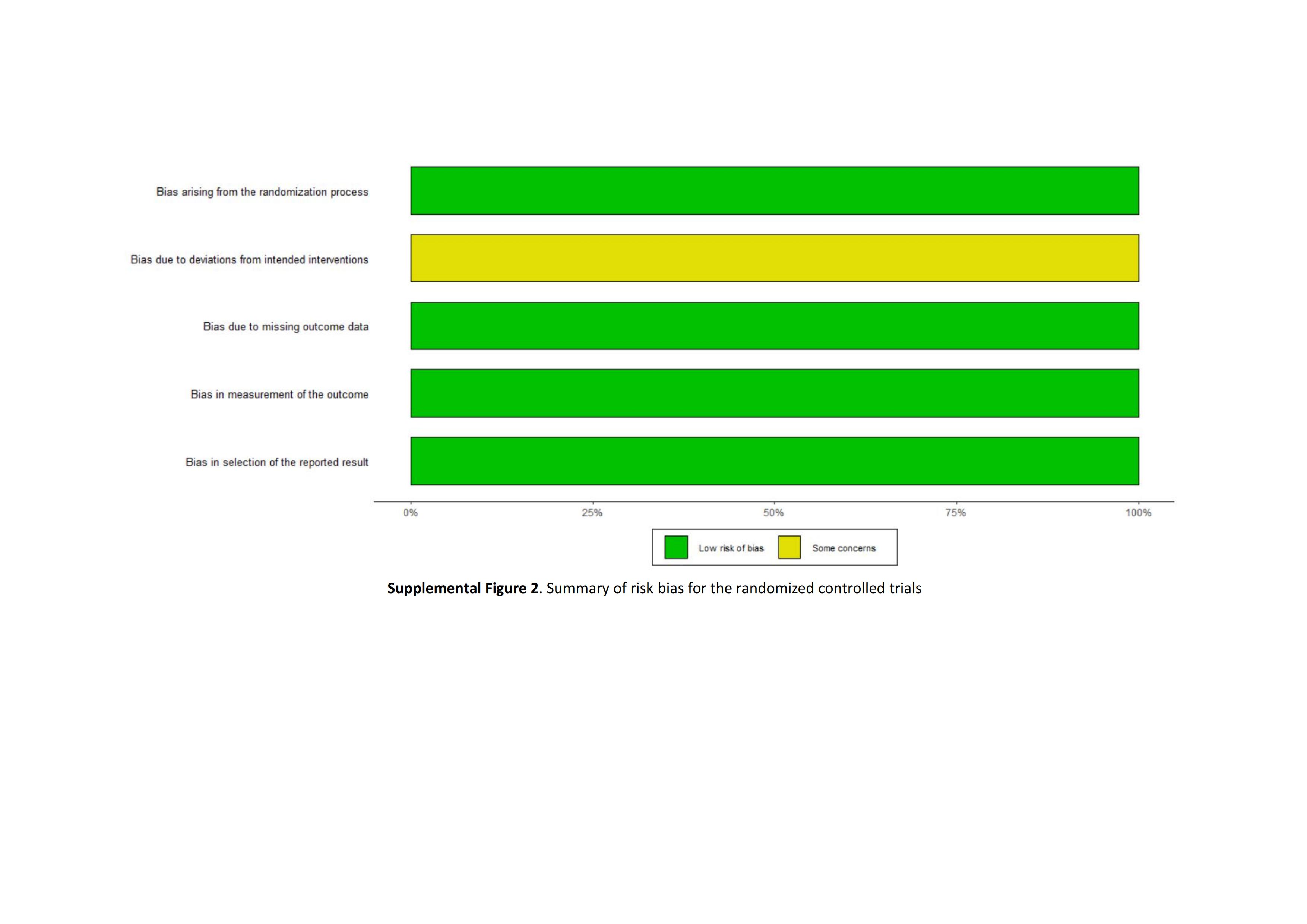

Supplement: Supplementary file 3 [file Image_2.jpeg]
